# Simulation Analysis of the Influence of Nozzle Structure Parameters on Material Controllability

**DOI:** 10.3390/mi11090826

**Published:** 2020-08-31

**Authors:** Huanbao Liu, Guangming Zheng, Xiang Cheng, Xianhai Yang, Guangxi Zhao

**Affiliations:** 1 School of Mechanical Engineering, Shandong University of Technology, Zibo 255000, China; zhengguangming@sdut.edu.cn (G.Z.); chengxiang@sdut.edu.cn (X.C.); zgx2019@sdut.edu.cn (G.Z.); 2 Analytical Testing Center, Shandong University of Technology, Zibo 255000, China; yxh@sdut.edu.cn

**Keywords:** 3D bioprinting, nozzle, orthogonal experiment, numerical simulation

## Abstract

With the evolution of three-dimensional (3D) printing, many restrictive factors of 3D printing have been explored to upgrade the feasibility of 3D printing technology, such as nozzle structure, print resolution, cell viability, etc., which has attracted extensive attention due to its possibility of curing disease in tissue engineering and organ regeneration. In this paper, we have developed a novel nozzle for 3D printing, numerical simulation, and finite element analysis have been used to optimize the nozzle structure and further clarified the influence of nozzle structure parameters on material controllability. Using novel nozzle structure, we firstly adopt ANSYS-FLUENT to analyze material controllability under the different inner cavity diameter, outer cavity diameter and lead length. Secondly, the orthogonal experiments with the novel nozzle are carried out in order to verify the influence law of inner cavity diameter, outer cavity diameter, and lead length under all sorts of conditions. The experiment results show that the material *P* diameter can be controlled by changing the parameters. The influence degree of parameters on material *P* diameter is shown that lead length > inner cavity diameter > outer cavity diameter. Finally, the optimized parameters of nozzle structure have been adjusted to estimate the material *P* diameter in 3D printing.

## 1. Introduction

The report shows that there is a demand of two-million organ transplants every year in China, but only 1% patients can get the right donor, most of them lose their lives because they do not have the matched replacement organ. The tissue and organ made by three-dimensional (3D) printing technology can provide more suitable donors for the patients, which is expected to solve the problem of donor shortage [1,2,3,4].

**Figure 1:**
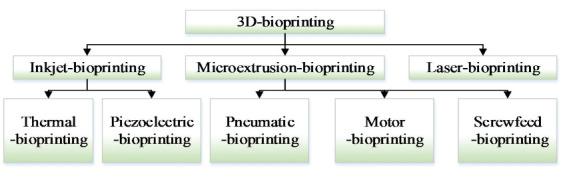
Three-dimensional (3D) bioprinter classification.

3D bioprinting has rapidly emerged as one of the modern tissue engineering technology that could potentially reconstruct the organ-like structures (with or without cells) [5,6], which is useful in treating organ diseases. Despite the great benefits and flexibility in printing a wide range of bioinks [7], there are many restrictive factors (such as nozzle structure, printing resolution, cell viability, etc.) to limit the application of 3D printing in the field of organ regeneration [8,9]. Many attentions have been attracted to improve the 3D bioprinting performance, all kinds of 3D bioprinting methods have been developed, which mainly include inkjet-bioprinting [10,11], microextrusion-bioprinting [12,13,14], and laser-bioprinting [15,16,17]. Figure 1 shows the detailed classification. In 3D bioprinting, it is necessary to keep layer-by-layer precise distribution of biomaterials and living cells, which can fabricate tissue or organ with spatial control of the placement of functional components. Several approaches (biomimicry, autonomous self-assembly, and mini-tissue building blocks) are applied to fabricate the complex tissue and organ. With the application of these methods in tissue engineering, several challenging technologies are proposed by many researchers [18,19,20]. One challenge is to adapt 3D printing technology designed to print inactive material (molten plastics and metals) to the printing of active biomaterials. Another further challenge is to reproduce the complex microarchitecture with multiple cell types in sufficient resolution to satisfy biological function [21].

Many research teams have adopted the above 3D printing strategies to fabricate organ-like structures with deposition modeling methods [22,23]. A controllable mechanism of materials has been explored to realize the controllability of material printing [14]. Delrot P. et al. [24] have used Laser-induced flow focusing to controllably generate viscous microdroplets up to 210 mPa.s. In those methods, the vessel-like structures have poor modeling effect and material accumulation phenomena. Therefore, three-dimensional axisymmetric flow-focusing was applied by GAO Q. et al. [25] to fabricate vessel-like structures, which proved that flow-focusing method can improve the cell survival rate. Meanwhile, Liu, H. et al. [26,27] have adopted the flow-focusing method to obtain vessel-like structures and analyzed the synchronization among nozzle extrusion, nozzle speed, and rotating speed based on an extrusion-based 3D bioprinter, which could improve the modeling effect in this forming method. As mentioned before, it can be seen that the flow-focusing method can achieve better formation of tissue and organ structure.

Based on this, we have developed a novel flow-focusing nozzle to observe material distribution controllability, numerical simulation, and finite element analysis have been used to optimize the nozzle structure and further clarified the influence of nozzle structure parameters on material controllability.

## 2. Structural Analysis

**Figure 2:**
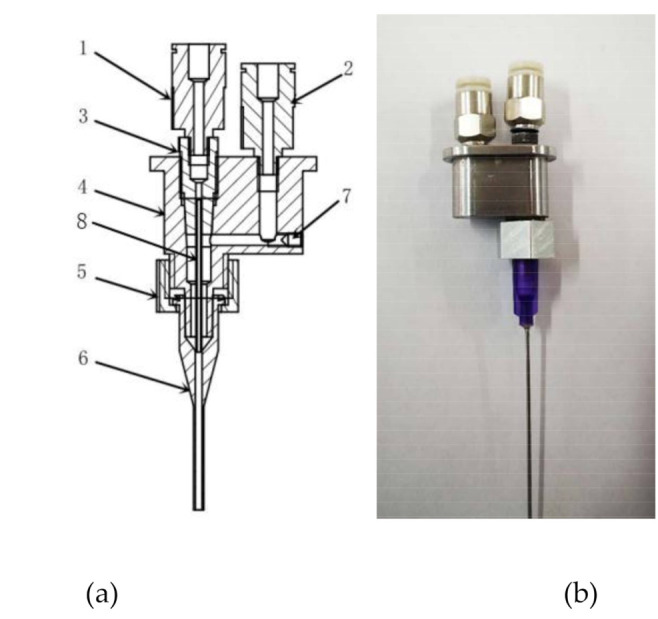
Optimized nozzle structure of 3D bioprinter. 1—Quick-connect (**a**), 2—Quick-connect (**b**), 3—Cone holder, 4—Nozzle body, 5—Set Nut, 6—pinhead, 7—Sealing screw, and 8—Capillary [26,27].

We have developed a novel flow-focusing nozzle to explore material distribution controllable mechanism (Figure 2), which mainly includes Quick-connect (a), Quick-connect (b), Cone holder, Nozzle body, Set Nut, Pinhead, Sealing screw, and Capillary. The Quick-connect(a) is connected to the inner cavity, the Quick-connect(b) is connected to the outer cavity, material *P* flows into inner cavity, material *Q* flows into outer cavity, under combined action of material *P* and material *Q*, the material *P* is extruded at a controlled diameter.

**Figure 3:**
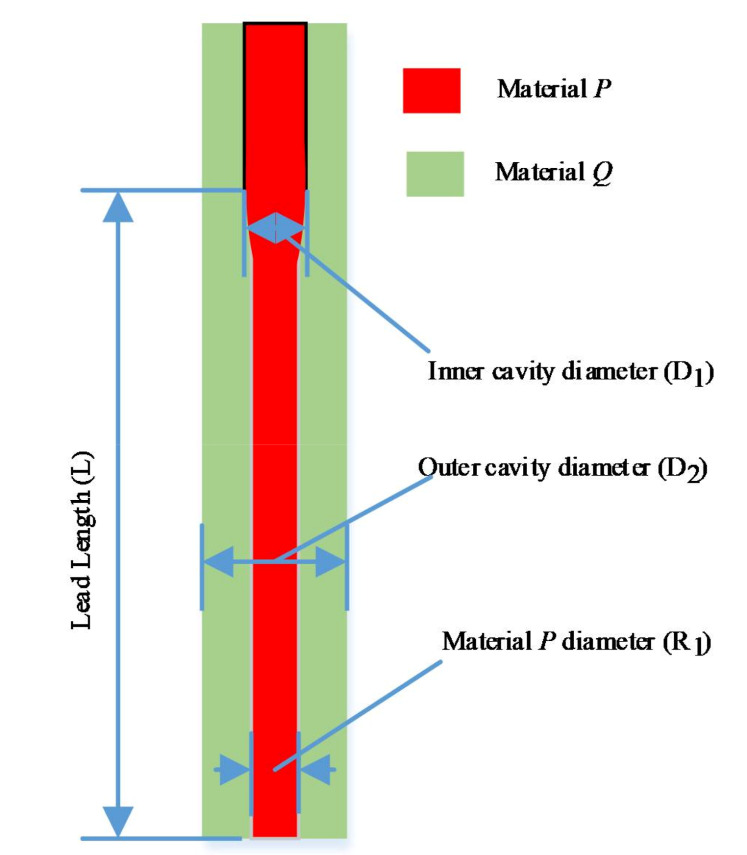
The evaluation index of 3D printing nozzle.

The orthogonal experiment method has been used to explore the material controllable mechanism in order to optimize the nozzle structural parameters and keep the nozzle printing effect in a peak state. The main evaluation index of 3D printing nozzle is the diameter of material *P* at the end of the nozzle (Figure 3). It is found that the following factors, such as the inner cavity diameter, the outer cavity diameter, the lead length, etc., have affected the printing and forming of the tissue and organ structure.

## 3. Simulation Analysis

Orthogonal experimental design is a multi-factor and multi-level design method. It selects some representative points from the overall test according to the orthogonality for the test. These representative points have the characteristics of uniform dispersion and neat comparability. In order to achieve material controllability, we have designed the orthogonal experiment method to optimize the inner cavity diameter D_1_, outer cavity diameter D_2_, and lead length L of 3D printing nozzle, the material *P* diameter R_1_ has been selected as the optimization index.

(a) Determination of test indicators

The inner cavity diameter D_1_, outer cavity diameter D_2_, and lead length L affect the diameter of material *P* at the end of nozzle. The combination of these factors also affects the quality of the printing.

**Figure 4:**
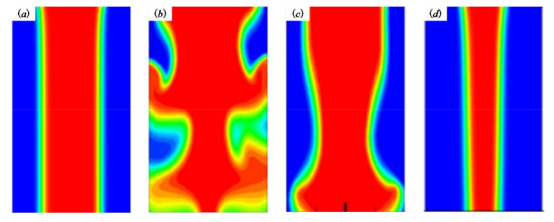
The phenomena occurred in the nozzle. (**a**) L is too short; (**b**,**c**) L is too short; (**d**) reasonable selection of the lead length L.

If the lead length is too short, the controllability of biomaterial extruded from the inner cavity is poor, which is not conducive to the control of the biomaterial diameter in the inner cavity and the subsequent printing and forming (Figure 4a). If the lead length is too long, the stability of the material *Q* encapsulation is poor due to the excessive pressure loss along the way (Figure 4b,c), which makes the phenomenon that the material cannot be extruded happen. If the extrusion state remains unchanged, the extrusion pressure of the material should increase, but the cells and other bioactive substances in the material *P* will be damaged under the condition of high pressure. Therefore, a reasonable selection of the lead length L will be more conducive to the extrusion of the pressure and the control of the inner cavity material (Figure 4d).

**Figure 5:**
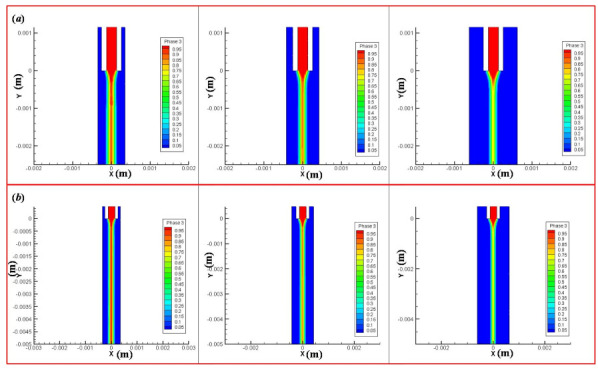
The influence of lead length and outer cavity diameter on the diameter of extruded material. (**a**) When the lead length is 2.5 mm, the change of extrusion material diameter under the outer cavity diameter 0.7, 0.84 and 1.25 mm. (**b**) When the lead length is 5 mm, the change of extrusion material diameter under the outer cavity diameter 0.7, 0.84, and 1.25 mm.

From Figure 5, when the lead length is 2.5 mm, the change of extrusion material diameter decreases with increasing the outer cavity diameter (Figure 5a). When the lead length is 5 mm, the change of extrusion material diameter decreases with increasing the outer cavity diameter (Figure 5b). We can see that the lead length L and outer cavity diameter D_2_ affect the extrusion diameter of the inner cavity biomaterial and the formability of fiber orientation. Therefore, the diameter of material *P* is taken as the experiment index of the material forming analysis.

(b) Experiment factors and determination of orthogonal experiment table

Inner cavity diameter D_1_, outer cavity diameter D_2_, and lead length L were chosen as the experiment factors. Each factor is described in detail below.

(1) Inner cavity diameter D_1_

It can be seen from the introduction of 3D biological printing nozzle that the diameter D_1_ of inner cavity is mainly used for extrusion control of internal biomaterials, and the size of inner cavity diameter directly affects the initial diameter of material extrusion. In this simulation test, the diameter of the inner cavity D_1_ is considered to be a dominant factor affecting the encapsulation state.

(2) Outer cavity diameter D_2_

The outer cavity diameter D_2_ can further control the output of the inner and outer materials, mainly including the thickness of material *P* and inner cavity material *P* diameter.

(3) Lead length L

Lead length L is the main factor that affects the encapsulation state. If the lead length L is too short, the controllability of the biomaterial extruded from the inner cavity is poor, which is not conducive to the control of the diameter of the biomaterial in the inner cavity and the subsequent printing and forming. If the lead length is too long, the encapsulation stability is poor, and even the material cannot be extruded because of the excessive pressure loss along the way. In this simulation test, lead length L is regarded as a factor that affects the encapsulation state. In this experiment, lead length L is set to three levels of 0 mm, 2.5 mm, and 5 mm.

To sum up, this orthogonal test is a three factor and three level test; the specific factors and levels are shown in Table 1.

The inner cavity diameter D_1_, outer cavity diameter D_2_, and lead length L are replaced by A, B, and C, respectively. The horizontal values of different factors are shown in Table 1. The orthogonal test table is designed by design-express software. According to the orthogonal test table, the simulation analysis is adopted ANSYS-FLUENT software to simulate and analyze the diameter of biomaterials in different situations, and the results are shown in Table 2.

## 4. Results

(1) Range analysis of test data

The simulation experiments are carried out in order to determine the influence degree of each parameter on the extrusion diameter (the primary and secondary factors). The test data have been processed according to the orthogonal experimental method, the extreme analysis results that represents the maximum range of parameter value are shown in Table 3. Where K_i_ is the sum of the test results corresponding to the factor at level I, the parameter K_i_ (av) is the mean value, R is the Range, which is the difference value between the maximum value and the minimum value under that factor.

Through the analysis results of the above simulation experiment, it can be concluded that factor C, namely the lead length, has the greatest influence on the extrusion diameter of the inner cavity biomaterials, followed by the nozzle inner cavity diameter, and finally the outer cavity diameter. The main reason for this phenomenon is that, with the increase of the lead length, the outer cavity material maintains a stable force on the inner cavity material, and the extrusion amount of the inner cavity material will shrink, which will lead to a reduction of the extrusion diameter.

(2) Variance analysis

The experiments have used the method of variance analysis to calculate and analyze the experimental results in order to make up for the shortcomings of the direct analysis method. The so-called variance analysis method distinguishes the difference between the experimental results caused by the change of factor level (or interaction) and the experiment results caused by the error fluctuation; the analysis results are shown in Table 4.

From the results of variance analysis, we can see that the inner cavity diameter D_1_, the outer cavity diameter D_2_, and lead length L have significant influence on the molding diameter of the inner cavity diameter, the inner cavity material diameter changes with the change of each factor level.

**Figure 6:**
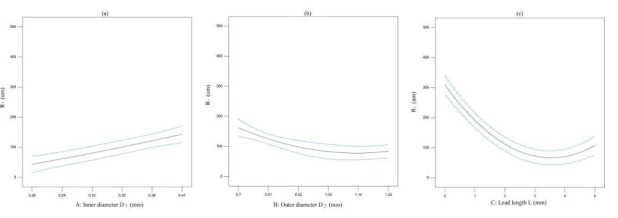
The effect of one factor for the diameter of biomaterial. (**a**) inner cavity diameter, (**b**) outer cavity diameter, (**c**) lead length.

**Figure 7:**
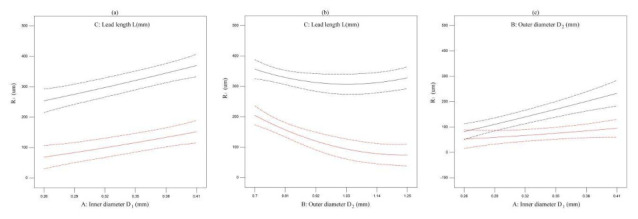
The effect of multifactor interaction for the biomaterial diameter. (**a**) interaction term (AC), (**b**) interaction term (BC), (**c**) interaction term (AB).

The effect of one factor for the diameter of extrusion biomaterial have been analyzed, as shown in Figure 4. The diameter of extrusion biomaterial increases with the increasement of inner cavity diameter (Figure 6a), the diameter of extrusion biomaterial increases with the increasement of outer cavity diameter (Figure 6b), the diameter of extrusion biomaterial decrease first and then increase with the increasement of lead length (Figure 6c). The effect of multifactor interaction for the biomaterial diameter has been shown in Figure 7. The interaction term (AC, BC) of factors has no significant influence, but the interaction term (AB) has significant influence on the material *P* diameter of biomaterials.

**Figure 8:**
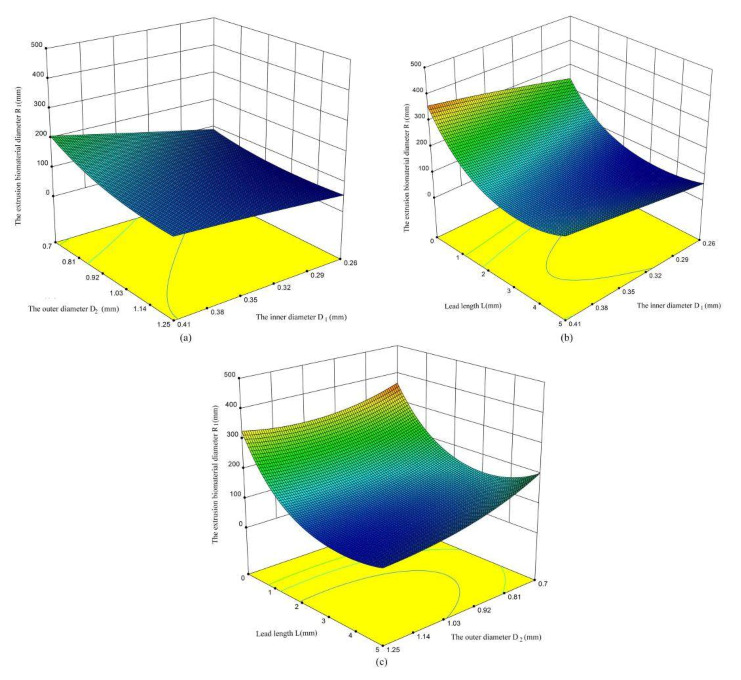
The influence of various factors on the extrusion biomaterial diameter. (**a**) the influence between outer diameter and lead length on R_1_; (**b**) the influence between lead length on and inner diameter on R_1_; (**c**) the influence between inner diameter and outer diameter on R_1_.

According to the analysis presented in Figure 6, the influence of various factors on the extrusion diameter of biomaterials is obvious. The extrusion diameter R_1_ of biomaterials increases with the increasement of the inner diameter of the nozzle (Figure 8b), and decreases with the increasement of the outer cavity diameter D_2_ of the nozzle (Figure 8a). With the increase of lead length L, there is a trend of decrease first and then increase (Figure 8c). The main reason for this trend is that the lead length is too long, the pressure loss along the lead length is large, and the phenomenon of material diffusion appears.

## 5. Discussion

Based on the advantages of flow-focusing method in tissue and organ regeneration, the novel nozzle has been developed to explored the biomaterial controllable mechanism. The main contents are as follows:

(1) A novel flow-focusing nozzle with encapsulation function has been developed. ANSYS-FLUENT software has been used to optimize the 3D bioprinting nozzle. The results show that changing the nozzle structure parameters can effectively control the diameter of material *P*, which further improves the encapsulation demand of 3D bioprinting nozzle, realizes the controllability of the material *P*, and improves the interchangeability of the nozzle.
(2) Through theoretical research and numerical simulation analysis, the controllable mechanism of material printing is studied under the pressure range 0–1 MPa. The experimental results show that the diameter of inner cavity biomaterial will decrease with the increase of the pressure in the outer cavity, and the shear force of outer fluid to the inner fluid will increase, resulting in the increase of the stress on the material *P*.
(3) Through the orthogonal test and analysis of the novel nozzle structure, it is determined that the main factors affecting the extrusion molding of the nozzle material include the inner cavity diameter D_1_, the outer cavity diameter D_2_ and lead length L. Based on the results of orthogonal test analysis, it is shown that the primary and secondary order of influencing factors on the molding effect is lead length L > inner cavity diameter D_1_ > outer cavity diameter D_2_. The extrusion diameter of material *P* can be better controlled by controlling the lead length and the inner cavity diameter.

In conclusion, we presented a novel flow-focusing nozzle, which can achieve material controlled distribution under the action of multi-parameter coordination. The significant results can not only achieve high controllability of biomaterials, but also lay the foundation of machine learning based simulation into 3D bioprinting and modelling of next generation nanoscaffolds and organs [28], which has the potential to provide better advantage to nanomedicine and medicine in general.

## Figures and Tables

**Table 1:** Factors and levels of orthogonal test.

Factor	Horizontal		
	1	2	3
Inner diameter D_1_ (mm)	0.26	0.33	0.41
Outer diameter D_2_ (mm)	0.7	0.84	1.25
Lead length L (mm)	0	2.5	5

**Table 2:** Orthogonal test results.

Orthogonal Test	A	B	C	R_1_ (Um)
1	0.33	1.25	5	86
2	0.33	0.84	2.5	110
3	0.41	1.25	2.5	95
4	0.33	0.84	2.5	110
5	0.26	0.84	0	260
6	0.33	0.7	0	330
7	0.41	0.84	5	200
8	0.33	1.25	0	330
9	0.33	0.84	2.5	110
10	0.26	1.25	2.5	55
11	0.41	0.84	0	410
12	0.33	0.84	2.5	110
13	0.26	0.7	2.5	103
14	0.41	0.7	2.5	--
15	0.33	0.7	5	205
16	0.26	0.84	5	80
17	0.33	0.84	2.5	110

**Table 3:** Extreme analysis.

Experiment Results	A	B	C
K_1_	498	638	1330
K_2_	1501	1500	803
K_3_	705	566	571
K_1(av)_	129.5	212.67	332.5
K_2(av)_	166.78	166.67	100.375
K_3(av)_	235	141.5	142.75
Range R	105.5	71.17	232.125
Major factor→Minor factor	C→A→B		

**Table 4:** Variance analysis.

Variance Analysis	Variance Source	Freedom	Square Sum	Mean Square	F Value	*p* Value	Significance
1	A	1	21.82	21.82	25.11	0.0002	significant
2	B	1	14.6	14.6	16.81	0.0008	significant
3	C	1	88.94	88.94	102.36	<0.0001	Extremely significant
4	AB	1	1.22	1.22	1.41	0.0203	significant
5	AC	1	0.24	0.24	0.28	0.3392	non-significant
6	BC	1	5.34	5.34	6.15	0.0133	significant
7	A^2^	1	0.076	0.076	0.088	0.7577	non-significant
8	B^2^	1	2.1	2.1	2.42	0.0340	significant
9	C^2^	1	71.77	71.77	82.6	<0.0001	Extremely significant
